# Neurocognitive and psychological symptoms in post COVID-19 patients (PASC24): prospective cohort study protocol

**DOI:** 10.1136/bmjopen-2026-116689

**Published:** 2026-07-17

**Authors:** Adrian Meehan, Zbigniew Dzialanski, Hans Hjelmqvist, Per Julin, Per Thunberg, Yvonne Freund-Levi

**Affiliations:** 1 School of Medical Sciences, Örebro University, Örebro, Sweden; 2 Department of Orthopedics, Örebro University Hospital, Örebro, Sweden; 3 Örebro University Hospital, Örebro, Sweden; 4 Department of Anaesthesiology and Intensive Care, Örebro University Hospital, Örebro, Sweden; 5 Department CLINTEC, Karolinska Institutet, Stockholm, Sweden; 6 Department NVS, Karolinska Institutet, Stockholm, Sweden; 7 Center for Experimental and Biomedical Imaging in Örebro, Örebro University Hospital, Örebro, Sweden; 8 Department Clinical Science and Education Internal Medicine KI Södersjukhuset at Karolinska Institutet, Karolinska Institutet, Stockholm, Sweden; 9 Department Clinical Trials, Örebro University Hospital, Örebro, Sweden

**Keywords:** Post-Acute COVID-19 Syndrome, Brain, Cognitive dysfunction, Magnetic Resonance Imaging, Digital Technology

## Abstract

**Abstract:**

**Introduction:**

Knowledge about the late effects of COVID-19, or so-called post-acute sequelae of COVID (PASC), and its pathogenesis is still very limited. The purpose of this observational study is to chart late neurocognitive effects of the COVID-19 infection. The aim is to increase knowledge of the underlying biological processes and describe how biomarkers correlate with degree of symptoms, mapping their role as diagnostic markers over time (24 months).

**Methods and analysis:**

This prospective observational cohort study is longitudinal and monitors patients with neurocognitive and psychological symptoms after COVID-19 infection in order to analyse the impact on the brain. For these purposes, we introduce a digital neuropsychological platform (Mindmore). We also aim to analyse potential diagnostic and prognostic markers from blood and cerebrospinal fluid and use MRI of the brain. We will measure the patients’ return to work and studies as well as any impact on their ADL functions and quality of life. The ongoing study aims to follow-up to 100 study participants with PASC for 24 months at three visits: baseline, 12 months and 24 months. The findings of the study can be used as guidance for future policy management in healthcare and may lead to a possible curative or symptom-relieving treatment.

**Ethics and dissemination:**

This study was approved by the Swedish Ethical Review Authority (diary number: 2020–0604 (main application), 2021–03205, 2023–00023-02, 2024–00426-02 (amendments)). Participants will be required to provide informed consent. The results of this study will be reported in peer-reviewed journals and presented at scientific conferences.

**Trial registration number:**

NCT06298006.

**STRENGTHS AND LIMITATIONS OF THIS STUDY**

- A study population that reflects the population of a well-defined, demographically diverse area in Sweden.
- Large amount/number of analysed biomarkers, including biomarkers from cerebrospinal fluid, that are difficult to obtain and therefore rarely investigated.
- Long follow-up period, 24 months.
- No control group built into the study design, but normative data for neurocognitive testing are available and will be used.
- Relatively low number of participants, which can possibly make statistical analysis of subgroups difficult.

## Background

 The WHO reports that since the outbreak of Severe Acute Respiratory Coronavirus 2 (SARS-CoV-2) in China in December 2019, over 778 million individuals have been infected, and over 7 million (7 098 155 as of 21–07-2025) deaths are confirmed associated with coronavirus disease 2019 (COVID-19).^1^ Before the initialisation of widespread vaccination programmes, many patients had severe respiratory difficulties and were in need of hospitalisation.^2^ However, the majority of patients have mild symptoms of SARS-CoV-2 including fever, coughs and fatigue, but most are, however, asymptomatic.^3^ Other commonly reported acute symptoms include sore throats, headaches, conjunctivitis and medical issues concerning the gastrointestinal tract.^4^ It has been noted that a number of patients have prolonged or, indeed, persistent symptoms including dyspnoea, fatigue, postexertional malaise and cognitive impairment requiring reduced working hours in almost half and inability to work in 22% of patients.^5 6^ Studies have shown that a significant proportion of patients who have a history of COVID-19 infection might show *neurological symptoms* such as headache, fatigue, epileptic seizures, impaired consciousness and ataxia; *psychiatric conditions* such as depression and anxiety and PTSD symptoms as well as *cognitive* impairment.^6 7^ Thus far, over 200 symptoms related to post-COVID-19 syndrome have been reported and affect almost all human physiological systems.^7 8^ The number of individuals reporting continued symptoms beyond 12 weeks is not insignificant and has led the National Institute for Health and Care Excellence (NICE) to term the condition ‘post-Covid-19 syndrome’ (PCC),^9^ while the term ‘long-covid’ (LC) is also widely used in research.^10^ Post-acute sequelae of COVID (PASC) occur in an estimated 10–30% of non-hospitalised patients and about twice as often in those who were hospitalised.^11^ In early phases of the pandemic, Hedberg *et al* reported a 4.8% hospitalisation rate among individuals testing positive for COVID-19 in Stockholm.^12^ Even with cautious estimations, it is approximated that worldwide as many as 65 million people may suffer from long-term symptoms from COVID-19 and cases are predicted to increase.^10 11^

PASC is hitherto neither fully characterised nor understood, making it a challenging condition to provide adequate medical management but which impacts seriously the activities of daily living (ADL) in those affected.^8^ PASC may lead to lengthy and recurrent periods of sick leave, costly for both the individual and for society at large.^13^ Subramanian *et al* report that PASC is associated with gender, ethnicity, socioeconomic status and a comorbidity burden.^14^ Since many related symptoms are self-reported, this imposes particular demands on the clinician to objectify findings. In a Swedish study, Wahlgren *et al* describe that cognitive impairment, sensorimotor symptoms and fatigue were the most prevalent even after 24 months follow-up with no difference between hospitalised and non-hospitalised patients.^15^ Cognitive symptoms have been further confirmed in a large community sample, irrespective of COVID-19’s duration.^16^ Furthermore, in a subgroup, white matter lesions were a common finding in MRI scans, which is also confirmed in other radiology studies.^15 17 18^

At present, there is an urgent need for objective biomarkers to assess and independently differentiate symptoms related to PASC from other morbidities in order to establish and provide appropriate rehabilitation alternatives. Increasingly, studies indicate immune activation that occurs during a moderately severe to severe COVID-19 infection and is similar to the one seen after hypoxic brain injury.^3 19^ This activation can be correlated with levels of cytokines and, for example, neurofilament light (NfL). NfL is an intermediate filament important for maintaining axonal stability and growth.^20^ Axonal damage results in the leakage of NfL into the cerebrospinal fluid (CSF), making it a marker for ongoing neuroaxonal degeneration. Another genetic biomarker: ApoE e4 genotype, long known to be associated with Alzheimer’s disease, has also been linked to the increased risk of developing a more serious COVID-19 infection, independent of pre-existing dementia, cardiovascular disease and type 2 diabetes.^21^

Another area of increasing clinical interest is gut bacterial microbiome in COVID-19, which is described as having decreased diversity and richness and persistent bacterial microbiome dysbiosis even after disease resolution.^22^ The gut mycobiome in COVID-19 is characterised by increased faecal fungal load and increased beta-diversity (more heterogeneous), and it is unstable over time and persistently altered after disease resolution. With this in mind, the present prospective study aims to collect clinically significant demographic, medical, neurocognitive data in patients with documented COVID-19 infection and to explore correlations between inflammation, neurocognitive findings, and the impact on the individual patient’s neurocognitive function and quality of life.

## Methods

### Study design

The current study is a prospective cohort study which we have named PASC (post-acute sequel of COVID-19) 24. The study is exploratory in nature as the subject (neurocognitive symptoms after COVID-19 infection) is relatively new. No treatment will be given during the study period. Therefore, the research team has chosen not to perform randomisation nor blinding.

**Figure 1:**
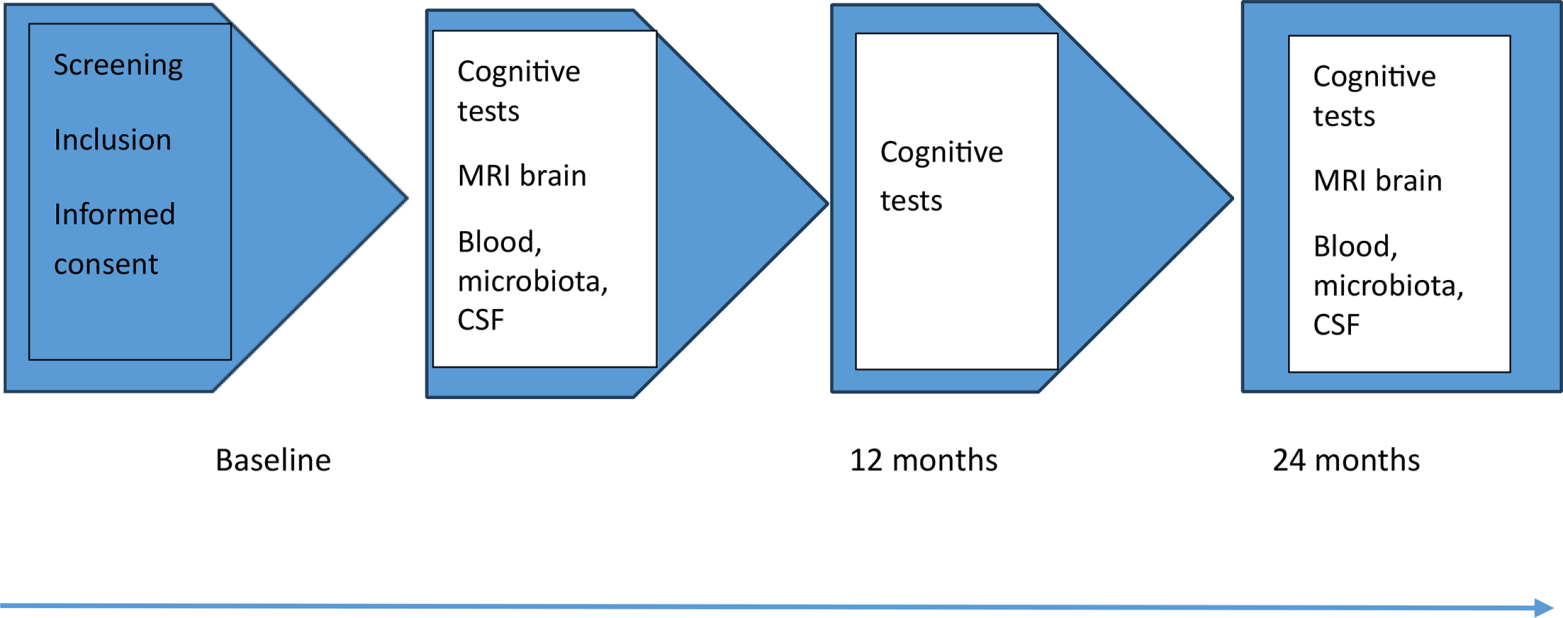
Flow diagram of recruited patients throughout the study. CSF, cerebrospinal fluid.

PASC 24 will be assessing and following recruited patients over a period of 24 months (figure 1). Three specific points of evaluation are planned, namely at baseline, after 12 months and 24 months. Recruitment began in 2024, and data collection is currently estimated to be completed by the end of 2026.

### Sample size calculation

The aim is to offer participation in the study to all patients in Örebro County who meet the inclusion criteria. Therefore, the sample size calculation has not been rigorously applied.

However, we have performed calculations based both on previous estimates of the frequency of PASC^10^ and on statistical considerations regarding future analyses. In both cases, we have concluded that a group of approximately 50–100 participants will be possible to recruit and provides space for meaningful analysis. The ethical permission applies to up to 100 participants.

### Study population (sample recruitment)

Participants in the PASC 24 study will principally be recruited primarily from Örebro County but may also be recruited from other areas of Sweden. There are approximately 300 000 inhabitants in Örebro County where the prevalence of SARS-CoV-2 is comparable to other parts of Sweden. A multidisciplinary conference (MDC) will ensure that the referred patients will have hitherto been given adequate and optimal care provision in Örebro County’s primary healthcare system (online supplemental file 1).

The overarching purpose is to detect and describe changes in physical, mental and cognitive function; daily activities and time-to-return to work in patients after experiencing moderate to severe COVID-19 infection. All patients will be living in the community and will be referred through their respective health centres to the MDC at the Department of Rehabilitation at the University Hospital Örebro with several health specialists, including cardiologists, geriatricians, neurologists and general practitioners.

All patients will be prescreened according to their premorbid cognitive and mood status by the PI (YVF specialist in psychiatry and geriatrics), so we can clearly indicate their premorbid status before inclusion in the study. Inclusion will be based on the definition from WHO (post COVID-19 condition (long covid)) concerning post covid syndrome, where the condition is characterised by a range of symptoms which usually start within 3 months of the initial COVID-19 illness and last at least 2 months. We will examine thoroughly patients’ medical records and check for a positive PCR test or positive antibodies in blood tests before inclusion.

Demographic data will be retrieved from medical records as well as in connection with inclusion: gender, age, medical diagnoses, medications, ethnicity, education, employment and degree of sick leave. Basic medical examination will be performed at inclusion: blood pressure, waist circumference, weight, BMI, and smoking habits (table 1).

**Table 1:** Sociodemographic and medical details identified in recruited patients in PASC 24-study

Variable	Variable
Age	Years
Sex	Man/Woman
Language	Swedish/other
Tobacco	Never/Previously/Occasionally/Daily
Social status	Living alone/Living with partner
Occupation	Employed/unemployed/pensioner
Education	Secondary or vocational/Upper secondary/university
Land of birth	Sweden/other
Certified sick-leave	>3 months
Certified sick-leave	Asthma/COPD/cancer/diabetes/heart insufficiency/hypertension/orthostatism/ hypercholesterolaemia/ neurological disorder/hypothyroidism/other
COVID-19 symptoms	Organ/specific symptoms
Smell/taste dysfunction	Remaining symptoms/previously

The *inclusion criteria* include COVID-19 infection confirmed by positive laboratory test (PCR test for SARS-CoV-2) taken during the acute infection, residual neurocognitive symptoms more than 3 months after the initial COVID-19 infection and an age of more than 18 years. Full-spectrum COVID severity cases will be included.

The *exclusion criteria* are severe cancer, dementia and ongoing alcohol or drug abuse. Included participants will then undergo baseline evaluation (figure 1). First when patients are included and have signed the patient consent form, they will then be tested using the neuropsychological tests tested via the digital platform Mindmore AB (www.mindmore.com).^23^ Mindmore is CE marked, approved, and purchased by the Research Centre of Region Örebro County. Trial participants may withdraw from the study at any time. Patients will receive no monetary compensation for study participation.

### Variables studied

A specialist physician (ZD) will perform all the clinical assessments when the patients are included, in part, to guarantee a stringent approach to clinical evaluations of participants. In addition to demographic data and medical examination, individual breathing function will be measured through peak expiratory flow (PEF) and muscular function and stamina by performing the 60-s chair-stand test.^24^ Furthermore, the CGI scale will be used for assessing clinical disease severity.

At both baseline and after 24 months, blood tests and lumbar puncture will be performed and faecal samples procured. Tables2  3 show the variables which will be measured and analysed. Most blood tests will be analysed locally at the clinical laboratory at the University Hospital in Örebro, Sweden. In order to analyse biomarkers in plasma, serum and CSF, samples will be stored at Örebro Biobank for later analysis. Cytokines (NGF, BDNF, IL-6, −8, −10, −12 and TNF-a) and ACE-2 in blood will be analysed with Olink Signature Q100, ApoE with TaqMan Allelic Discrimination technology, kynurenic acid with UPLC/MS/MS and GC/MS, NfL with enzyme-linked immunosorbent NF-Light kits. With the same methods, the following variables will be analysed from CSF: NfL, kynurenic acid, ACE-2 and markers of dementia (T-tau, Ph-tau, Amyloid-β 40/42). Microbiota samples will be gathered from the study participants in order to further examine the gut-brain connection.

**Table 2:** Serum and cerebrospinal fluid (CSF) variable, and faecal samples measured at baseline and after 24 months respectively

Variable	Values/Information
Serum/blood	Hb, MCV, WBC, 5-parts diff
	Glucose, HbA1c
	CRP-high sensitivity
	Albumin, AST, ALT, CDT, GT
	Na, K, Creatinine/e-GFR
	TSH/T4, T3
	Total cholesterol, HDL-C, LDL-C
	Vitamin D
	NGF, BDNF, IL-6, −8, −10, −12, TNF-a
	ACE-2, kynurenic acid, NfL, ApoE4
CSF	β-amyloid 42, Amyloid β 42/40
	Tau protein, P-Tau
	Neurogranin
	Neurofilament
	ApoE4
Faeces	Microbiota composition
Physiological outcome variables (also taken at 12 months visit)	Puls, blood pressure, weight, height, waist circumference, BMI, PEF, 60 s chair-stand test

ALT, Alanine Aminotransferase; AST, Aspartate aminotransferase; CDT, Carbohydrate-deficient transferrin; CSF, Cerebrospinal fluid; GT, Gamma-glutamyl transferase; Hb, Haemoglobin; HDL-C, High-density lipoprotein cholesterol; LDL-C, Low-density lipoprotein cholesterol; MCV, Mean Corpuscular Volume; NfL, Neurofilament light chain; PEF, Peak expiratory flow rate; WBC, white blood count.

**Table 3:** Neurocognitive and psychiatric measurements assessed at baseline and after 12 and months respectively

Variable	Values/Information
Neurocognitive function: attention and processing speed	Trail making test part A (TMT-A)
	Symbol digit modalities test (SDMT)
	Simple reaction time test
Memory:	Rey auditory verbal learning test (RAVLT)
	CERAD word list learning test (CERAD)
	Corsi block-tapping test (CORSI)
Language:	Token test
	Boston naming test (BNT)
	FAS word fluency test (FAS)
	Cube drawing test
Visuospatial function:	Clock drawing test
Executive function:	Trail making test part B (TMT-B)
	Silent Stroop test
	Complex reaction time test
Psychological function	MADRS, MADRS-S
	MFS
	PCL-5
	PSS-14
	CGI-S
Overall health status and well-being	EQ-5D-5L
	EQ VAS

Participants’ neurocognitive functions will be assessed objectively with multiple tests facilitated through a digital platform provided by Mindmore AB by using two test batteries. All tests are based on validated neuropsychological rating scales. Raw neurocognitive test results will be compared with normative data for the Swedish population and converted to norm-calibrated scores: z-scores.^25^ The testing will provide a broad and deep neurocognitive profile of the patient and will assess the domains of attention and processing speed, memory, executive functions, visuospatial function and language.^25^ *Attention and processing speed* will be assessed by means of the Trail Making Test part A^26^ and Symbol Digit Modalities Test.^27^ *Memory* will be assessed by the 15-word Rey Auditory Verbal Learning Test (RAVLT),^28 29^ a 10-word auditory and visual learning test (CERAD Word List Learning Test)^30^ and a spatial memory test.^31^ *Language* will be assessed using the Token Test^32^ to measure auditory comprehension, the Boston Naming Test (BNT) – 15 words^33^ and, finally, the FAS Word Fluency Test.^34^ *Visuospatial functions* will be determined through the Cube Drawing Test^35 36^ and the Clock Drawing Test.^37 38^ Finally, *executive functions* will be assessed primarily through the TMT part B, measuring mental flexibility, and an in-house test of complex reaction time^23^ to measure information processing; additionally, a silent Stroop Test^39 40^ will measure concentration effectiveness. Task engagement and performance-based validity will be checked using two tests: Token Test and RAVLT, embedded into test batteries.

Complementary psychological examinations will be conducted at baseline, and at follow-up after 12 and 24 months, respectively. All tests used are validated instruments and translated into several languages. Patient-reported measurements will be obtained and registered digitally (Smart-Trial) in connection with the visit, which, if needed, will include an interpreter. Given the high symptom overlap between PTSD and common diagnoses such as depression, anxiety and ADHD, it is important for accurate diagnosis to assess the relationship between current symptoms and therefore we will use the Post-Traumatic Stress Scale (PTSD) PCL5.^41^

The Mental Fatigue Scale (MFS), which was constructed to capture the impact of mental fatigue on various life situations,^42^ is a self-assessment tool consisting of 14 defined symptoms of mental fatigue. Within each of the areas, the person rates the degree of impact on a 7-point scale, with support text provided for every other scale step. A total score is calculated. A score above 10.5 is considered mental fatigue of such a degree that it negatively affects functioning in daily life. Premorbid cognitive status will be checked using the Mini-mental state Examination Test.^43^

For premorbid depressive status mood examination, we will use the Montgomery Åsberg Test.^44^ The Montgomery-Åsberg Depression Rating Scale^44^ assesses and tracks changes in clinical depressive symptoms and is an interview-based instrument with 10 items corresponding to the main depressive symptoms, where each individual item is answered on a six-point scale depending on the severity of the depressive symptoms. The scale is specifically designed to track changes in depressive symptoms. For analysing the premorbid stress levels, we will use the PSS 14.^45^ Activity of daily living will be analysed using the EQ−5D- 5 L.^46^ To rule out symptoms related to Attention Deficit Hyperactivity Disorder (ADHD), we will use the screening battery of ASRS.^47 48^

Brain MRI will be performed at study inclusion and at the end of the study period, no later than 24 months from inclusion. The MRI system is a 3.0 Tesla scanner, and the examination protocol includes the following imaging acquisitions: structural T1 weighted (T1W), fluid attenuated inversion recovery (FLAIR), T2 weighted (T2W), susceptibility weighted (SWI), quantitative susceptibility mapping (QSM), diffusion tensor imaging (DTI), arterial spin labelling (ASL) and resting state functional MRI (rsfMRI). A structural report based on radiological findings will be provided by a neuroradiologist. The images acquired from the MRI examinations will enable us to do the following analyses: voxel-based morphology (VBM), functional and structural connectivity, cerebral blood flow (CBF), presence and extent of cerebral microbleeds in combination with QSM.

### Statistical analysis

Data will be processed using SPSS (version 22). Before performing statistical analysis, all data will be scrutinised for quality, distribution and missing data. Descriptive statistics (means, medians, frequencies and percentages) will be used to characterise the data. Differences between independent groups will be analysed using independent t-test for continuous outcomes and Pearson’s χ^2^ test or Wilcoxon Signed Rank Test will be used to find associations between two categorical/ordinal variables. Regression or repeated measures ANOVA (or other applicable method) will be used to test neuropsychological change over time (z-score change) to find the independent variables significant to the outcome.

Statistical significance will be set at p<0.05.

### Ethics approval and dissemination

The study will be performed according to the Guidelines of Good Clinical Practice and will follow the ethical principles for medical research involving human subjects according to the Declaration of Helsinki, adopted by the 18th General Assembly of the World Medical Association (WMA 1964). The study has been approved by the Swedish Ethical Review Authority, diary number: 2020–0604 (main application), 2021–03205, 2023–00023-02, 202400426–02 (amendments). The study has been registered at ClinicalTrials.gov with the number NCT06298006. All study participants will provide informed consent and will be informed of their right to opt out of the study at any time. The data will be handled in pseudonymised form, with the code key kept by the registry holders.

Separate manuscripts will be written on several of the secondary aims, and these will also be submitted for publication in peer-reviewed journals. After completion of the study, and after publication of the primary outcomes, data requests may be applied for directly from the researchers.

### Patient and public involvement

Throughout the preparation period and initial stages of recruitment the research group has actively engaged with the public and patient groups through, for example, public meetings with a registered national patient society for COVID patients, through podcasts, and articles in the local press. Results from this study are planned to be disseminated through patient societies at the end of the study period.

## Discussion

Long-covid or post-acute sequelae of COVID-19 (PASC) is thought to be a common consequence of SARS-CoV-2 infection, but the condition is not fully understood. To the best of our knowledge, this is the first Swedish study aimed at comprehensively describing and analysing biomarkers, radiology findings, neurocognitive testing and quality of life outcomes in patients reported to have PASC. Long-term studies have hitherto shown inconsistent results in the study of adults.^49 50^ It is suspected that PASC may lead to longstanding effects, including structural and functional brain adaptations.^51 52^

In addition, studies have shown biomarkers monocyte/chemokine signatures in CSF and MRI abnormalities. These findings suggest that the prognosis for brain-fog following COVID-19 correlates with myeloid-related chemokine and interferon-responsive genes.^53^ The need for such studies is imperative since the impact on society in terms of distress for the affected individuals and the risk for long-term sick leave is potentially considerable.

One of the main difficulties in constructing knowledge and evidence concerning PASC is due to the nature of the studies available. Despite considerable international collaboration and open access to COVID-19 studies, there exists a high level of heterogeneity in study designs, follow-up periods and the strategies for surveying.^10^ Currently, the absence of specific biomarkers for the diagnosis of PASC creates immense challenges for the medical practitioner to provide correct help for the patient.^54^

PASC-24’s study protocol is both hypothesis-driven and explorative in its construction. For instance, levels of kynurenic acid increase in patients with major depression and kynurenine:tryptophan ratios are seen to be altered in patients infected with COVID-19.^3 55^ Some observational studies indicate changes in gut microbiome composition in patients with long-term complications of COVID-19 where, for example, butyrate-producing bacteria, including *Bifidobacterium pseudocatenulatum* and *Faecalibacterium prausnitzii*, show the greatest inverse correlations with post-COVID infected patients at 6 months.^56 57^ This current study plans, therefore, to analyse the composition of intestinal flora and a range of inflammatory biomarkers and analyse changes over longer time in order to investigate any correlation with persistent symptoms.

There are many conflicting issues concerning PASC. This includes the role of common biomarkers in the determination of risk for developing PASC or indeed, the role of stress and pre-infection frailty.^58 59^ Additionally, there is an impending need for long-term studies to further explore and analyse the interrelationship between PASC and the commonly reported ‘brain-fog’ correlated to cerebral structural changes.^60^,^62^

Our current study may have some limitations. Acceptance for the PASC condition has diminished regionally, nationally and internationally creating difficulties in both recruitment and handling of patients.^63^ This has very real consequences in terms of resource allocation devoted to this patient group. There are clear methodological challenges in diagnosis differentiation, as noted in many studies.^64^ Predicting the dropout rate is extremely difficult as we are not aware of any other study results with repeated lumbar puncture in patients with

PASC, though another similar study reported attrition rates of up to 40%.^65^ Finally, it is our intention that by using a comprehensive, multi-modular assessment of individuals with symptoms related to the debut of SARS-CoV-2 infection, we might further describe and understand the symptomatology, risk and prognostic factors of PASC and how these individuals perform over a 2-year period of follow-up. This might contribute to the establishment of adequate systems of patient management.

## Supplementary material

10.1136/bmjopen-2026-116689 online supplemental file 1
