# Supplementary figures and images for: Neurocognitive and psychological symptoms in post COVID-19 patients (PASC24): prospective cohort study protocol
Source: BMJ Open. 2026 Jul 17;16(7):e116689. doi: 10.1136/bmjopen-2026-116689 (PMC13384184; doi:10.1136/bmjopen-2026-116689)

# Project I: Material and Methods

## Recruitment of study participants

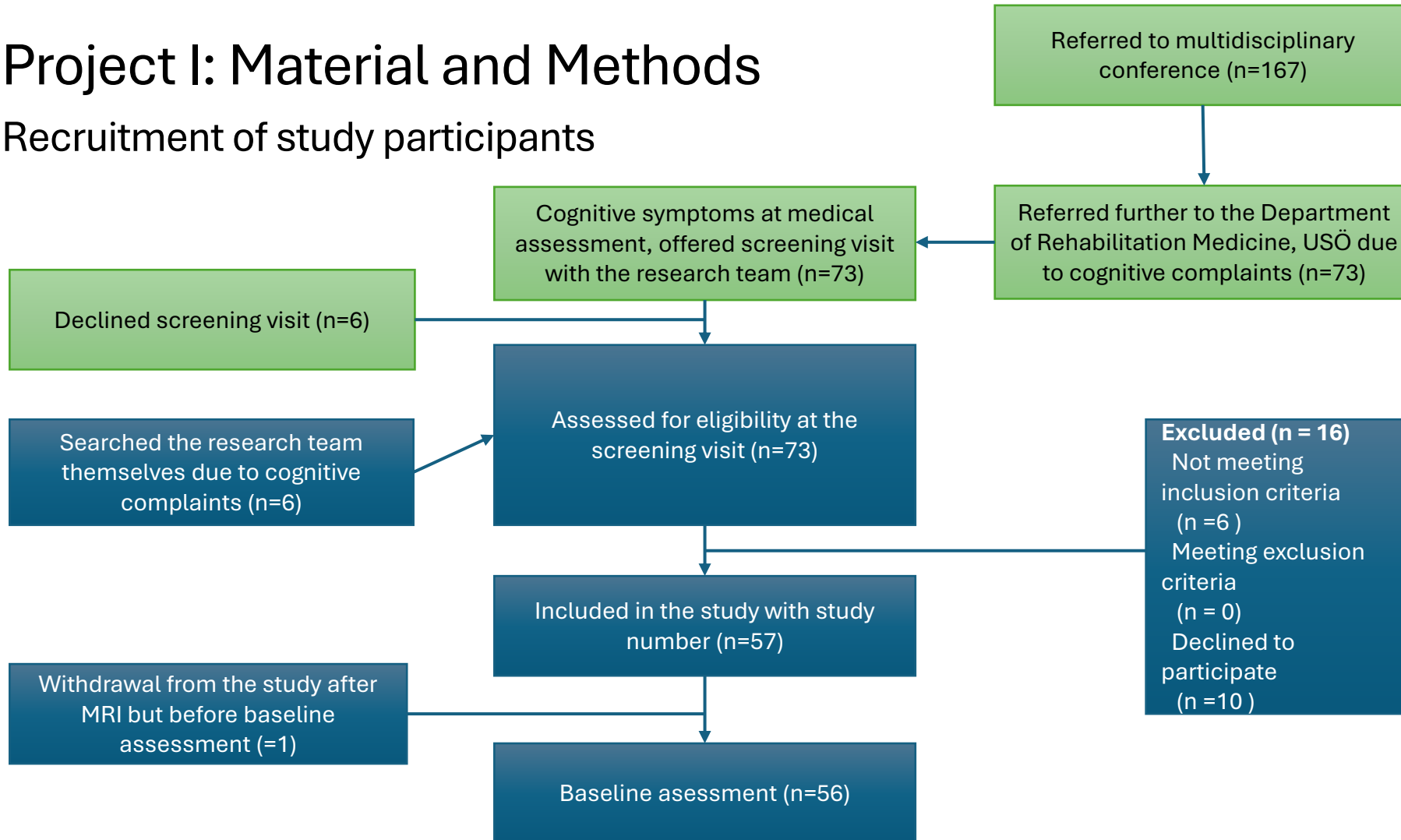

Supplement: online supplemental file 1 [file bmjopen-16-7-s001.pdf]
